# Comparing Growth Models Dependent on Irradiation and Nutrient Consumption on Closed Outdoor Cultivations of *Nannochloropsis* sp.

**DOI:** 10.3390/bioengineering12030272

**Published:** 2025-03-10

**Authors:** Tiago Taborda, José C. M. Pires, Sara M. Badenes, Francisco Lemos

**Affiliations:** 1 A4F—Algae for Future, Campus do Lumiar, Estrada do Paço do Lumiar, Edif. E, R/C, 1649-038 Lisbon, Portugal; sara.badenes@a4f.pt; 2 Cerena, Instituto Superior Técnico, Av. Rovisco Pais 1, 1049-001 Lisbon, Portugal; francisco.lemos@tecnico.ulisboa.pt; 3 LEPABE—Laboratory for Process Engineering, Environment, Biotechnology and Energy, Faculty of Engineering, University of Porto, Rua Dr. Roberto Frias, 4200-465 Porto, Portugal; 4 ALiCE—Associate Laboratory in Chemical Engineering, Faculty of Engineering, University of Porto, Rua Dr. Roberto Frias, 4200-465 Porto, Portugal

**Keywords:** microalgae, photobioreactors, pilot-scale reactors, growth kinetics, model comparison

## Abstract

Microalgae offer tremendous industrial possibilities for their ability to grow rapidly and capture CO_2_ from the atmosphere. The literature contains many models for predicting microalgae growth in lab-scale reactors. However, there exists a gap in the application of these models in outdoor pilot-scale closed photobioreactors. This work proposes a methodology for constructing models for this type of reactor. These models were constructed based on the existing literature, then trained and tested using a dataset of ten cultivations of *Nannochloropsis* sp. Four models were tested: a model based on a Monod-like equation (Model M); a model based on a Haldane-like equation (Model H); a model based on an exponential equation (Model E); and a model considering both irradiation and the effect of nitrate on the culture using the Droop model (Model D). Model H had the best overall performance, with a global root mean squared error (RMSE) of 0.296 kg^1/2^ m^−3/2^; Model M and Model E had RMSE values of 0.309 and 0.302, respectively. Model D performed the worst, with an RMSE of 0.413. Future work should involve applying the same methodology to new cultivations of the same or different species and testing more complex models capable of better explaining the data.

## 1. Introduction

Microalgae are a diverse group of photosynthetic single-celled eukaryotic organisms. They are found in almost all aquatic environments, from freshwater to marine or even hyper-saline water, in extreme pH ranges (very acidic or very alkaline), and at extreme temperatures. Microalgae are a promising source of several organic products, like high-quality vegetal oil (which can be used for biofuel), livestock feed, and other high-valued bioactive metabolites, such as pharmaceuticals, cosmetics, and even antibacterial, antifungal, and anticancer compounds [1].

Microalgae offer several advantages over other sources for these specific products. They grow quickly and reach high areal productivity compared to conventional land-based plants. Microalgae also do not require arable land to be grown and require less freshwater, meaning they do not compete with conventional crops. They can also capture CO_2_ from polluting industries, such as cement production [2] and flue gases from fossil fuels [3] after appropriate pre-treatment.

Over the past few decades, microalgae production has been subject to intense research and large-scale investments. These originally involved biofuel production, but more recently, have been focused on niche products such as pharmaceuticals [1], since these are expected to be the most lucrative microalgae-based products. However, the technical feasibility, economics, and environmental benefits of large-scale algal cultivation are still subject to research [4]. One of the ways to potentially solve these issues is by constructing robust models of the systems of interest. These models may allow for better monitoring of the system’s inputs (such as nutrient intake) and predict future cultivations under similar conditions. Due to being much easier to control and monitor, closed photobioreactors are ideal systems to accurately model despite requiring significantly more complex models than other reactor types.

There is a significant repertoire of microalgae growth models reported in the literature; however, the direct application of models to outdoor pilot-scale microalgae cultivations in closed photobioreactors is relatively sparse, mostly focusing on small, indoor cultivations. Fernández et al. [5] created a model based on solar irradiation to estimate the growth rate of the species *Phaeodactylum tricornutum* in tubular photobioreactors with air-lift pumps (0.220 and 0.050 m^3^), which was based on the day of the year, the geographical location, and the reactor geometry, taking into account photo-limitation and photoinhibition. They reported a coefficient of determination (R^2^) of 86% using a Monod-like hyperbolic model for the effect of photo-limitation. The same group later created a similar model with fence-type photobioreactors (2.5 m^3^) inside a greenhouse growing *Scenedesmus almeriensis* [6]. The growth model used considers reactor geometry. Although the authors did not report a value for the goodness of fit for the biomass, they reported that the other parameters they simulated (oxygen concentration and carbon dioxide) had good fits. Ippoliti et al. [7] constructed a growth model to predict the indoor and outdoor growth of *Isochrysis galbana* in the same facility and reactor type as that used by Fernández et al. [5]. Their model also predicted oxygen production using variables like temperature, dissolved oxygen, pH, and irradiance. They reported a good fit of the model that was fitted using indoor data, to outdoor data later obtained.

There is currently a gap in the literature concerning the application of this type of model of microalgae growth to outdoor photobioreactors. This work is an attempt at filling this gap by applying these growth models to different pilot-scale (approximately 1 m^3^) cultivations of the microalgae *Nannochloropsis* sp. in closed photobioreactors (PBRs) with different configurations but under similar growth conditions, the same geographical location, and under natural solar irradiation, considering the nutrient influx of the system, the volume of the bioreactors, and the frequency and quantity of the renovations. The light and nutrient-dependent growth models are based on those reported in the literature for indoor and outdoor cultivations. The application of these models to real-life pilot cultivations is novel and absent in the literature. Cultivations of *Nannochloropsis* sp. was chosen for this study because of its potential interest in an industrial setting due to its ability to accumulate lipids when starved of nitrogen [8]. The constructed semi-empirical models will then be able to be used to predict the performance of future cultivations using similar growth conditions.

## 2. Materials and Methods

This work involves the modeling of ten cultivations of *Nannochloropsis* sp. performed by A4F—Algae for Future at its Lisbon Experimental Unit in Portugal over the years 2015 to 2020.

### 2.1. Bioreactor Configurations and Assays

**Figure 1:**
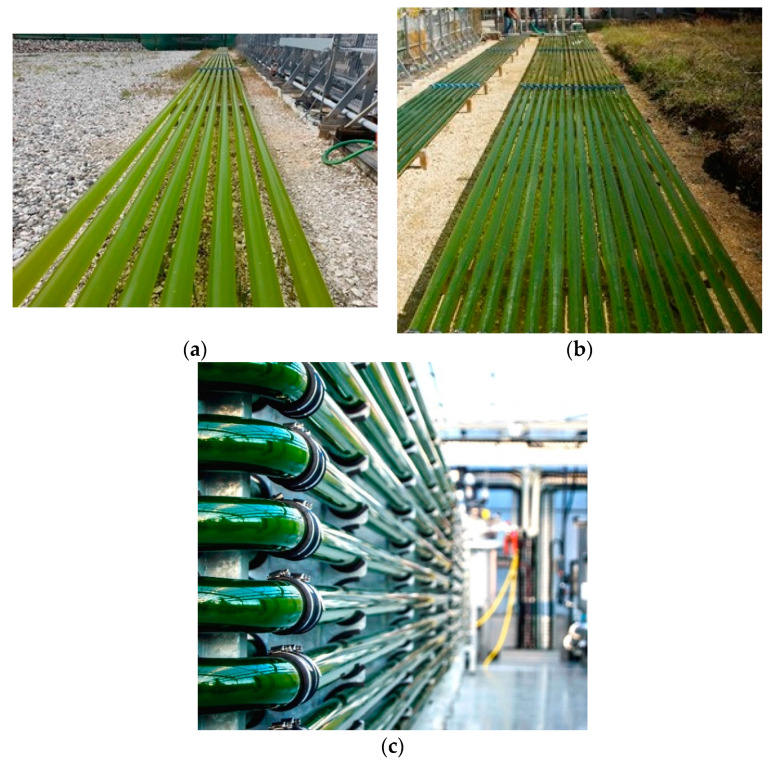
Photographs of the reactors used in this article. (**a**) U1 reactor; (**b**) U2 reactor; (**c**) M1 reactor.

Cultivations were done in three different bioreactor configurations, named U1, U2, and M1. The U1 and U2 reactors were unilayer horizontal tubular photobioreactors (UHT-PBR), with the U1 reactor having 8 lines of tubes and the U2 reactor with 16 lines of tubes; they were exposed directly to sunlight. The M1 reactor was a multilayer horizontal tubular photobioreactor (MHT-PBR) with 24 lines of tubes stacked in two columns of 12 lines of tubes each; it was placed inside a greenhouse and thus was not exposed directly to sunlight. The continuous recirculation velocity of the culture inside the tubes was between 0.7 and 1 m/s. A photograph of the three reactor types can be found in Figure 1.

All reactors had a cooling system that was activated whenever the internal temperature reached more than 30 °C to maintain that temperature or lower; this was achieved using a serpentine immersed in the air exchange chamber. *Nannochloropsis* sp. was cultivated using A4F artificial saltwater industrial medium (modified ESAW [9] with salinity ranging from 30 to 35 g/L and containing nitrogen, phosphorous, and other essential micronutrients). The reactors were run in a batch regime, with nutrients added as needed. Partial harvesting of the reactor contents (renovations) was done occasionally over the course of each assay. Only solar irradiance and non-limiting nutrient conditions were used for all assays. For all cultures, the pH was maintained at 8.0 through on-demand injection of pure CO_2_. The viability of the cultures and absence of contamination was checked via microscopic observation of samples of the reactor medium.

There are ten assays, named arbitrarily with the reactor type they were cultivated in and a number (for example, U1-1, U2-2, and M1-3). They were divided into two groups with five elements each: the assays used to fit the models (the training assays) and the assays used to test the models (the validation assays).

Each assay was assigned to the training or validation group based on the following criteria:

- Having the same number of assays in each group;
- Having a balance of the number of multilayer and unilayer assays in each group.

The codes given for each of the assays, along with their duration, the number of data points collected in each, and whether they are a training assay or a validation assay, are presented in Table 1. In total, there were 251 data points that were distributed between the training (150 data points) and validation (101 data points) datasets.

### 2.2. Measurements and Process Data

All reactors had the same parameter measurements and process data available: total culture volume (measured every day); harvest frequency and proportion, usually between 7 and 14 days and between 20 and 80% of the culture volume; dry cell weight, measured through optical density taken approximately twice per week; nitrogen concentration, measured approximately twice per week (using the APHA 2012 Section 4500 NO3-B method [9]); temperature of the reactor, recorded manually thrice per day at 9 am, 12 am, and 5 pm; and pH of the reactor, recorded manually thrice per day at 9 am, 12 am, and 5 pm. All these measurements were done with duplicates. The irradiation values used in the models were measured at the location of the reactors every 15 min by a weather station mounted on the site, allowing the consideration of variations in the weather conditions, such as overcast or cloudy days, which lowered the measured irradiation. Whenever irradiation data were unavailable, for example due to data corruption, data were obtained from the Photovoltaic Geographical Information System (PVGIS) [10] which has measurements available every hour. The details for the determination of the dry cell weight through the optical density is explained in Appendix A.

### 2.3. Data Processing and Model Construction

For each assay, the laboratory records were combined with data from the on-site weather station or PVGIS to generate an hourly dataset containing the key parameters required for the models. All the models tested were constructed in MS Excel using the data described above. All the models tested shared the same global parameters for all the assays used in that model. The biomass and nutrient concentrations measured at the beginning were used to start the simulation at time 0.

The models were fitted to the ensemble of data in the training set using the Solver add-on by minimizing the sum of squared residuals (*SSR*—see Equation (1)) of the dry cell weight by varying the growth model parameters.

(1) $$SSR=\sum_{i=1}^{n}{\left(y_{i}-{y_{i}}^{\prime }\right)}^{2}$$

where *y_i_* is the measured dry cell weight, and *y_i_’* is the calculated dry cell weight for that point in time.

Due to the short step used in the integration of the differential equations (1 h), the Euler method was considered adequately precise. The Runge–Kutta method of the 4th order was used to check the accuracy of the numerical integrations, but no appreciable difference was found between the two methods. Euler’s method also presents the advantage of being significantly simpler to implement and providing faster optimization procedures.

### 2.4. Models Tested

The main part of all models is the growth rate (*μ*, h^−1^) equation, which determines how light, nutrients, and the influence of the concentration of biomass affects the growth rate at any given step of the simulation. When a factor other than light was considered, the method used to balance the effects of all these influences was the multiplicative method, suggested by Darvehei et al. [11], as found in Equation (2).

(2) $$Μ=\mu_{max}\prod \mu_{i}$$

where *µ_i_* represents each term considered (light, influence of the concentration, and nutrients). Other recorded parameters, such as temperature and pH, were also considered, but their inclusion did not show any significant improvement in the models. Therefore, only light, nutrients, and biomass concentrations were considered for the modeling.

As described by Béchet et al. in their review article [4], growth models for photobioreactors can be divided into three main types: Type I models, where the rate of photosynthesis for the entire culture is exclusively a function of the incident light intensity reaching the external surface of the system; Type II models that account for the existence of light gradients in the system; and Type III models that consider the movement of the microalgae cells inside the reactor and its effects on the photosynthetic capabilities of the individual microalgae cell. For this work, only Type I models were considered. However, it was found that Type I models alone could not adequately explain the growth rate of cultures due to the fact that, on some occasions, high cell concentrations occur inside the reactor, leading to significant light attenuation. Therefore, a subtype of Type I models was considered. Here, it is assumed that in a turbulent, well-mixed culture, all cells are exposed to a certain amount of average incident light *I_av_* (W m^−2^ or mol_photons_ m^−2^ s^−1^) [12], which is a function of the incident light at the surface of the reactor and some attenuation function *f* that depends on the concentration of the biomass (Equation (3)).

(3) $$I_{cell}=I_{av}=I_{0}\cdot f(X)$$

The light attenuation can be determined using several different equations, but the simplest and most commonly used is the Beer–Lambert law (Equation (4)) derived for a homogeneous medium, with no scattering and at local thermodynamic equilibrium [13].

(4) $$I_{eff}\left(z\right)=I_{0}\exp\left(-E_{a}Xz\right)$$

with *I_eff_* (W m^−2^ or mol_photons_ m^−2^ s^−1^) being the light that effectively reaches the cells, *z* (m) the direction of light propagation (assuming the light propagates in only one direction), *X* (g L^−1^) the particle concentration, and *E_a_* (L g^−1^ m^−1^ or m^2^ g^−1^) the mass attenuation (or absorption) coefficient.

Models using the average light intensity can be described by the same equations as those using the incident light intensity, with the addition of a term depending on the Beer–Lambert law. Since the average light intensity inside a reactor is difficult to calculate, a simpler approach was taken, where the product of *E_a_* and *z* is considered to be a single constant which is termed *α*, or a Beer-Lambert-like constant (m g^−1^). *E_a_* is dependent on the specific species being considered, but it is considered a constant since the same species was used in all assays. Considering the photobioreactor tubes all have the same diameter and are oriented along the same cardinal directions, *z* is considered a constant that does not vary with the reactor type; *z* should vary with the position of the sun in the sky, which varies with the time of day and the day of the year, but it is assumed for these simple models that *z* is also a constant. Therefore, *α* is also a constant that will be a model parameter in all modeling approaches. It is assumed that all cells in the reactor are exposed to the same value as *I_eff_*, that is, the culture is well-mixed enough to prevent stratification of the reactor volume, creating light and dark regions.

All models assume 1st order growth, that is,

(5) $$\frac{dX}{dt}=\mu X-\delta^{D}\frac{V_{recirc}X}{V}$$

where *V* is the volume (m^3^), *V_recirc_* is the recirculated volume (in m^3^), and *δ^D^* is the Dirac delta.

Integrating this equation using Euler’s method and considering the renovations, Equation (6) is derived.

(6) $$X_{t+1}=X_{t}+h\mu X_{t}-\sum_{j}^{\cdots }{\delta^{K}}_{t,j}\frac{V_{recirc}X}{V}$$

where *h* is the integration step in hours, *V_recirc_* is the volume recirculated in the time period *j* (in m^3^), *δ^K^* is the Kronecker delta, *t* is a discrete variable with all the periods considered in the integration (in hours), and *j* is a discrete variable with all the periods where a renovation occurred (in hours).

#### 2.4.1. Model M

Model M uses a Monod-like equation for the effect of incident light, as suggested by Darvehei et al. [11] and Van Wagenen [14], and used by many other authors [15,16,17,18].

(7) $$\mu_{I}=\mu_{max}\frac{I_{eff}}{K_{S}+I_{eff}}$$

where *K_S_* is the half-saturation constant (W m^−2^). The parameters adjusted are *μ_max_*, *K_S_*, and *α*.

#### 2.4.2. Model E

Model E is an exponential model [19,20] with the following equation.

(8) $$\mu_{I}=\mu_{max}\left(1-exp\left(-\frac{I_{eff}}{\lambda }\right)\right)$$

where *λ* is the value of *I* for which µ=1−1eµmax ≈0.6321 µmax (W m^−2^).

Since the value of *λ* in the equation as formulated is not intuitive, by changing the base of the exponent to 2, a new parameter can be introduced, *λ*_2_, which is the value of *I* for which $µ=0.5\;{µ}_{max}$; that is, *λ*_2_ is the value of *I* for which the growth rate is half the maximum growth rate. It can be thought of as analogous to *K_S_* of the Monod model (although the shape of the curves is different). The final model, including *I_eff_*, is shown in Equation (9).

(9) $$\mu_{I}=\mu_{max}\left(1-2^{\left(-\frac{I_{eff}}{\lambda_{2}}\right)}\right)$$

The parameters adjusted are *μ_max_*, *λ*_2_, and *α*.

#### 2.4.3. Model H

Model H has an additional parameter to capture potential light inhibition. It is based on the work of Haldane [21] and its reparameterizations by Eilers and Peeters [22] and Bernard and Rémond [23]. All reparameterizations of this model have been used by several authors in their work [7,16,18,24,25,26,27,28,29].

However, it has been found that the original Haldane model’s parameters are arbitrary and do not represent physical constants, thus making it difficult to derive conclusions about the behavior of the culture. Both reparameterizations of the model were found to be inadequate for fitting, with Eilers and Peeters’ version having a potentially negative constant and Bernard and Rémond’s model raising numerical problems when used to fit the experimental data. Thus, a new model reparameterization is hereby proposed in Equation (10).

(10) $$\mu_{I}=\mu_{max}\frac{k}{k+{\left(I_{eff}-I_{opt}\right)}^{2}}\;k=2\gamma I_{eff}I_{opt}$$

with *I_opt_* (W m^−2^) being the value of irradiation for which the growth rate is maximized and *γ* a dimensionless shape factor. The derivation of this equation can be found in Appendix B. Mathematically, it can be shown that a value of *γ* equal to 1 reduces this model to that of Lee [16], shown in Equation (11).

(11) $$\mu_{I}=\mu_{max}\frac{I}{K_{1}+K_{2}I^{2}}$$

with *K*_1_ (W m^−2^) and *K*_2_ (m^2^ W^−1^) being its parameters. The parameters adjusted are *μ_max_*, *γ*, *I_opt_*, and *α*.

#### 2.4.4. Model D

Model D introduces the influence of nutrients. It is based on the 2015 work of Nikolaou et al. [30]. This model is based on three differential equations to calculate the substrate (*S*, g_N_ m^−3^), biomass concentration (*X*, g_C_ m^−3^), and the carbon-specific nitrogen quota (*Q*, g_N_ g_C_^−1^).

Because of the different reactor layouts, the equations used are simplifications of the ones presented in the original paper. Since harvesting occurs in discreet intervals, there is no dilution term. Likewise, endogenous respiration was not considered in these models. Therefore, the differential equation for the biomass is identical to the one presented in Equation (6), and the remaining equations are simplified to the ones presented in Equation (12).

(12) $$\left\{\begin{array}{c} \frac{dX}{dt}=\mu X\\ \frac{dS}{dt}=-\rho X\\ \frac{dQ}{dt}=\rho -\mu Q \end{array}\right.$$

where *ρ* is the nutrient uptake rate (g_N_ g_C_^−1^ h^−1^).

The growth rate was simplified when compared with the work of Nikolaou et al. It is a combination of the well-established Droop model [31] with a Monod-like influence for light, as shown in Model M.

(13) $$\mu =\mu_{max}\frac{I_{eff}}{K_{I}+I_{eff}}\left(1-\frac{Q_{min}}{Q}\right)$$

where *Q_min_* is the minimum nitrogen quota that still allows for growth (g_N_ g_C_^−1^).

The nutrient uptake rate is the same as that used by Nikolaou et al. [30].

(14) $$\rho =\rho_{max}\frac{S}{K_{sub}+S}\left(1-\frac{Q}{Q_{I}}\right)$$

where *ρ_max_* (g_N_ g_C_^−1^ s^−1^) is the maximal nutrient uptake rate, *K_sub_* (g_N_ m^−3^) is the half-saturation constant for the nutrient, and *Q_I_* (g_N_ g_C_^−1^) is the limit of the nitrogen uptake.

The fitting parameters are *μ_max_*, *K_I_*, *Q_min_*, *ρ_max_*, *K_sub_*, *Q_I_*, and *α*.

### 2.5. Comparison Metrics

To objectively compare the ability of the models to describe the experimental data, two objective metrics are used: the root mean squared error (RMSE) and the Akaike information criteria.

#### 2.5.1. Root Mean Squared Error (RMSE)

The RMSE is a regression evaluation method that considers the sum of squares and the number of data points, as well as the number of estimated parameters. The lower the RMSE, the better the fitting. The RMSE (kg^1/2^ m^−3/2^) is calculated using Equation (15).

(15) $$RMSE=\sqrt{\frac{\sum SSR}{n}}$$

where *SSR* is defined in Equation (1) and *n* is the total number of data points. The RMSE was calculated for both the training and validation datasets.

#### 2.5.2. Akaike Information Criterion

The calculation of the Akaike information criterion (AIC) follows the method presented by Burnham and Anderson [32]. The best model using this criterion is the one with the lowest value. Because the only important metric to compare AICs is using their differences, the values calculated are given as ∆*AIC* (differences in AIC) using Equation (16).

(16) $$∆AIC=2k+n\cdot ln(\sigma^{2})$$

where *k* is the number of parameters of the model, and *σ*^2^ is the variance, which is calculated using Equation (17).

(17) $$\sigma^{2}={RMSE}^{2}$$

### 2.6. Bootstrapping

To obtain information about the precision with which the parameters are estimated, bootstrapping was used to calculate the average and standard deviation of each parameter in each model. The method used is the one described in [33].

The new objective function is defined as a weighted least-square approach, as shown in Equation (18).

(18) $$SSR=\sum_{i=1}^{n}{w_{i}(y_{i}-f(y_{i}))}^{2}$$

where *w_i_* is the weight assigned to each data point. For each data point in the training set, a weight of 0, 1, or 2 was randomly assigned, with a uniform distribution. The objective is to replace one third of the data points from the original dataset with duplicates of points from the same dataset, thus preserving the total number of data points and the statistical characteristics of the error distribution in these points. The weight was multiplied by the squared error value for that data point, and the Solver add-on in MS Excel was run to minimize the modified error value. The Solver add-on was initialized with the parameter values equal to the original fitting (that is, with all the weights set to 1). This process was repeated 250 times for each model, using macros to allow the estimation of 250 values for the fitting parameters, which could then be statistically analyzed, and the average and standard deviations were computed.

## 3. Results & Discussion

### 3.1. Model Parameter Analysis

The parameters obtained for each model are as follows:

- Model M: *μ_max_* = 0.041 h^−1^; *K_S_* = 13.3 MJ m^−2^ day^−1^; and *α* = 1.645 m^3^ kg^−1^;
- Model H: *μ_max_* = 0.032 h^−1^; *I_opt_* = 41.4 MJ m^−2^ day^−1^; *γ* = 1.070; and *α* = 1.337 m^3^ kg^−1^;
- Model E: *μ_max_* = 0.033 h^−1^; λ_2_ = 9.933 MJ m^−2^ day^−1^; and *α* = 1.515 m^3^ kg^−1^;
- Model D: *μ_max_* = 0.082 h^−1^; *K_S_* = 63.3 MJ m^−2^ day^−1^; *α* = 1.003 m^3^ kg^−1^; *Q_min_* = 0.0025 g_N_ g_C_^−1^; *Q_max_* = 8 × 10^−6^ g_N_ g_C_^−1^ h^−1^; *K_sub_* = 0 g_N_ g_C_^−1^; and *Q_I_* = 3.98 g_N_ g_C_^−1^.

For Model H, the value of *γ* being approximately 1 implies that it is possible to simplify the model to that of Lee and eliminate one variable from the model without resulting in a significant loss of accuracy.

The values *K_S_* from Model M and λ_2_ from Model E are small and very close, which implies the culture achieved its maximum growth rate at low values of effective irradiation.

#### 3.1.1. Maximum Growth Rate

Boussiba et al. reported a maximum growth rate of 0.030 h^−1^ for *Nannochloropsis salina* [8]. Van Wagenen et al. reported a maximum specific growth rate of 0.054 h^−1^ for this species, with the relationship between growth and light intensity adjusting well to a Monod-type equation [14]. Huesemann et al. also reported the same 0.054 h^−1^ value for *Nannochloropsis salina* using an unspecified growth model [34].

The specific growth rates for Models M, H, and E fall within the range given in the literature. However, the maximum growth rate for Model D is significantly above the other models. This could be explained by the method used to weigh the contributions of the different limiting factors for growth (namely, the irradiation intensity and the effect of nutrients), which multiplied both factors together. This means that their positive or negative effects are compounded. The actual maximum achieved growth rate, as produced by Model D, is between 0.0321 and 0.0505 h^−1^, which is in line with the literature values.

#### 3.1.2. Half-Saturation Constant for Light

Van Wagenen et al. reported a *K_S_* of 37 μmol m^−2^ s^−1^ [14] for *Nannochloropsis salina*. This value is, however, not directly comparable to the *K_S_* values calculated for the models presented in this work because the units are in moles of photons and not energy. It is, however, possible to convert between the two values by considering the distribution of the wavelengths of light in the spectrum of sunlight. These calculations are explained in Appendix C. The calculated conversion factor is 0.2160 J μmol^−1^. Using this value, the converted values for *K_S_* are as follows:

- Model M: 61.6 μmol m^−2^ s^−1^;
- Model D: 293 μmol m^−2^ s^−1^.

As can be seen by these results, the *K_S_* values obtained in the models are higher than the *K_S_* reported for this species, and this value is higher for Model D than for Model M. A higher value of *K_S_* means the culture’s growth rate is lower for smaller irradiation values. The discrepancy between the values of *K_S_* for Models M and D is due to the higher value of *μ_max_* in Model D, which at low values of irradiation can account for the difference in the higher value of *K_S_*. The difference in species might also account for a small discrepancy in the values of *K_S_.*

#### 3.1.3. Beer–Lambert-like Constant

A Beer–Lambert-like constant was added to all models to account for the effect of light absorption in the cultures at higher concentrations. A value of 0 would imply no effect (the value of the exponential term would be 1); the higher this value, the more the effect of light attenuation inside the reactor. All models (except Model D) had this constant around 1.5 m^3^ kg^−1^, which implies there is, in fact, attenuation of light inside the reactor, with this effect being more significant for higher concentrations of biomass.

### 3.2. Model Fitting Analysis

**Figure 2:**
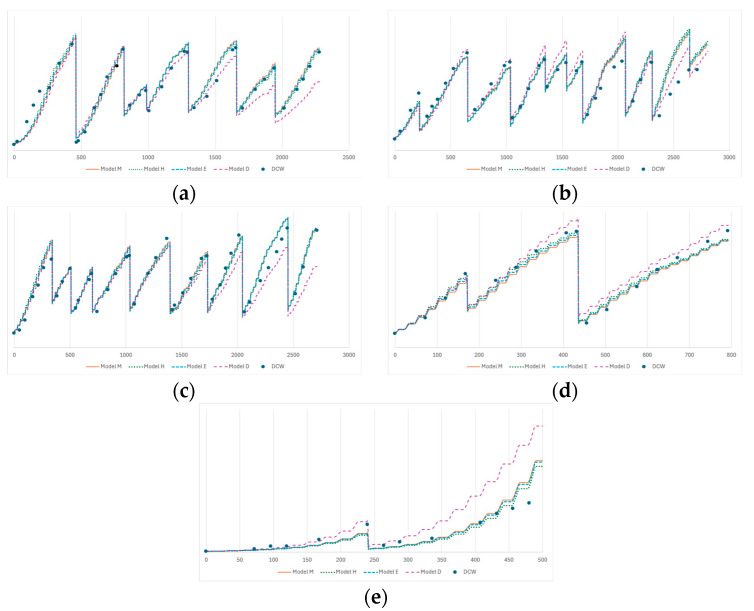
Results of the fitting of the models to the five training assays. The dots are the measured dry cell weights for each assay. The dashed lines are the fitted models. The *x*-axis is the time since inoculation (in hours); the *y*-axis is the biomass concentration (in kg m^−3^). (**a**) M1-2; (**b**) M1-4; (**c**) U1-1; (**d**) U1-2; and (**e**) U2-2.

The results of the fittings of the four models being considered can be found in Figure 2. The values of the RMSE of each model and assay can be found in Table 2.

Overall, the fitting of Models M, H, and E to the five training assays is very similar and a good match for the experimental data, with Model H having the best performance. This result implies that there is, in fact, an effect of light inhibition for higher irradiance values (above *I_opt_* = 41.4 MJ m^−2^ day^−1^). Model D shows a much higher RMSE, which arises from the poor fitting observed in most assays.

**Figure 3:**
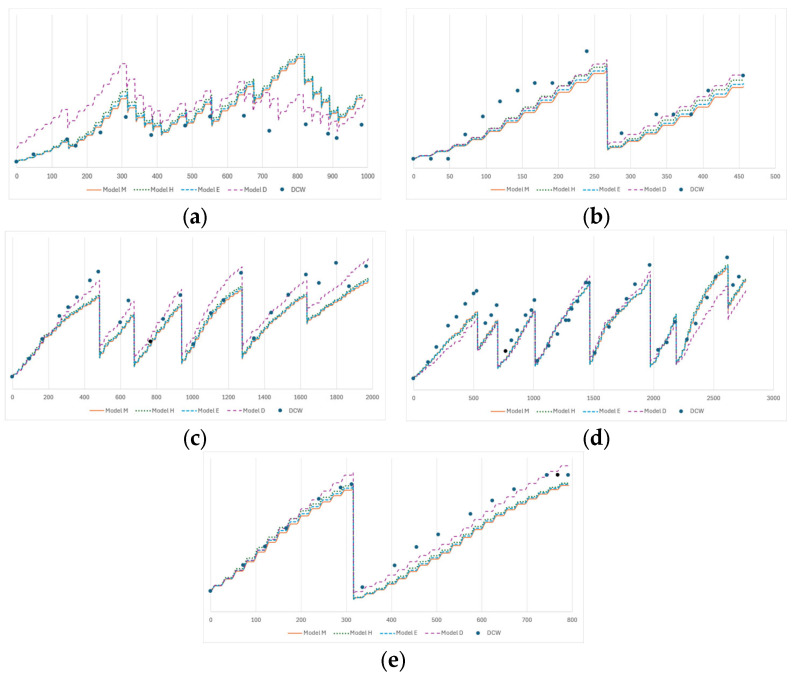
Results of the fitting of the models to the five validation assays. The dots are the measured dry cell weights for each assay. The dashed lines are the fitted models. The *x*-axis is the time since inoculation (in hours); the *y*-axis is the biomass concentration (in g L^−1^). (**a**) M1-1; (**b**) M1-3; (**c**) U1-3; (**d**) U2-1; and (**e**) U2-3.

The results of applying the models with the parameters previously obtained to validation assays can be found in Figure 3. A summary of the RMSE of each validation assay can be found in Table 3.

In general, like with the training assays, Model H once again performed the best, with the lowest RMSE, and Model D had the highest RMSE, mainly due to the poor adjustment to the M1-1 assay.

From these results, all models seem, in general, capable of explaining both the training and validation assays. Model H is overall the best at modeling both the training and validation assays.

### 3.3. Calculation of the Akaike Information Criterion (AIC)

The calculated values for ∆AIC, including all assays, were as follows:

- Model M: −694;
- Model H: −717;
- Model E: −707;
- Model D: −505.

Thus, according to the AIC, the best model of the four tested is, once again, Model H because it has the lowest ∆AIC, confirming the results that were obtained from the analysis of the RMSE.

### 3.4. Comparative Analysis of the Models

As shown in the previous sections, the four presented models showed good performance when fitted with the given dataset. However, it is also necessary to take a more holistic approach and interpret the validity of application of the models.

In terms of biological interpretability, all models were chosen based on pre-existing, well-tested models in the literature. The parameters of each model can be understood in terms of established physical and biological phenomena, like light inhibition, light attenuation inside the reactor, and nutrient limitation. Model H was a reparametrized version of the existing Haldane models to better showcase parameters that could otherwise be difficult to interpret.

The models presented are also generalizable, being applied to three different reactor configurations with no discernible differences between them. That said, only one species was considered for this article, which might limit its applicability to other species.

Models M, H, and E are robust, as they are not sensitive to large variation in the input conditions. They can withstand a wide range of input conditions and still perform as one would expect. Model D appears to be less robust, especially when the effect of nutrients is considered.

The models are also capable of explaining new data, as shown by the low RMSE values of the validation assays.

Finally, simpler models were shown to be as effective, if not more effective, at fitting the data, as Models M, H, and E (simpler models) performed better than Model D, and Models M and E (simpler models with just three parameters) were nearly as effective as Model H (which has four parameters).

### 3.5. Bootstrapping Statistics

**Figure 4:**
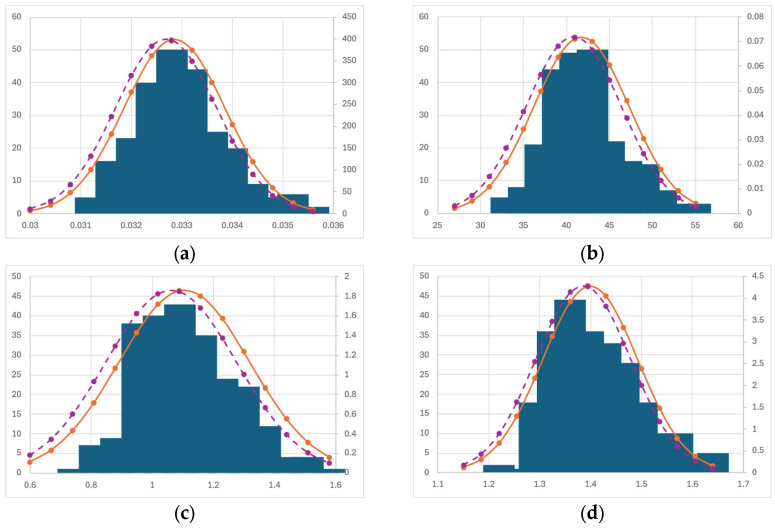
Histograms and the normal distribution for all the fitting parameters of Model H. The dashed lines correspond to the normal distribution when the median is taken as the mean value in the normal distribution. (**a**) *µ_max._* (**b**) *K_I._* (**c**) *γ.* (**d**) *α*.

**Figure A4:**
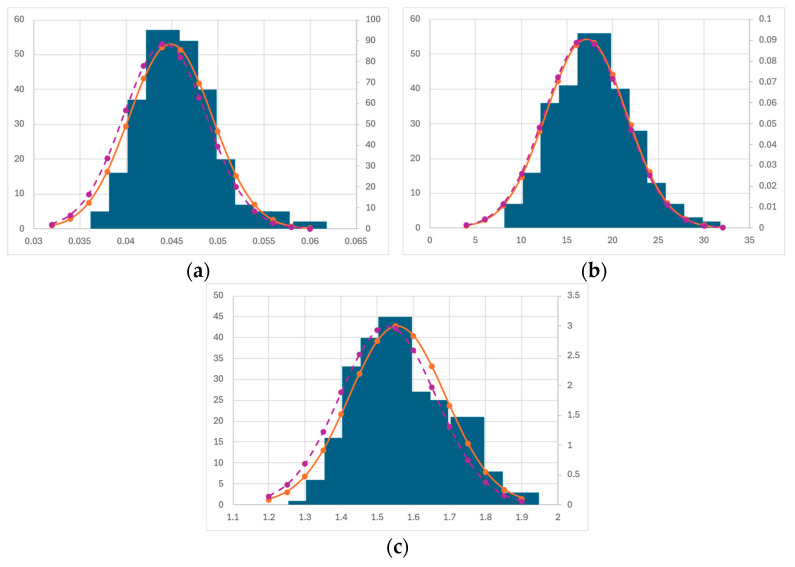
Histograms and the normal distribution for all the fitting parameters of Model M. The dashed lines correspond to the normal distribution when the median is taken as the mean value in the normal distribution. (**a**) *µ_max._* (**b**) *K_I._* (**c**) *α*.

**Figure A5:**
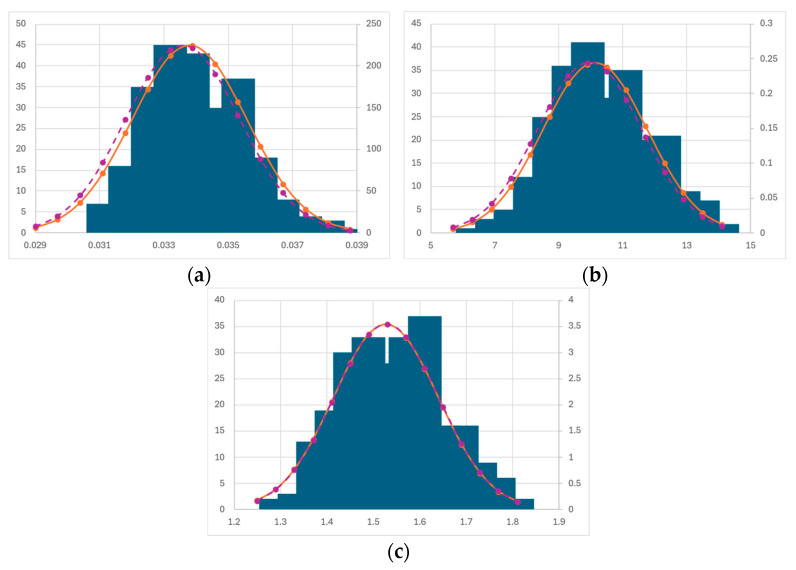
Histograms and the normal distribution for all the fitting parameters of Model E. The dashed lines correspond to the normal distribution when the median is taken as the mean value in the normal distribution. (**a**) *µ_max._* (**b**) *λ.* (**c**) *α*.

**Figure A6:**
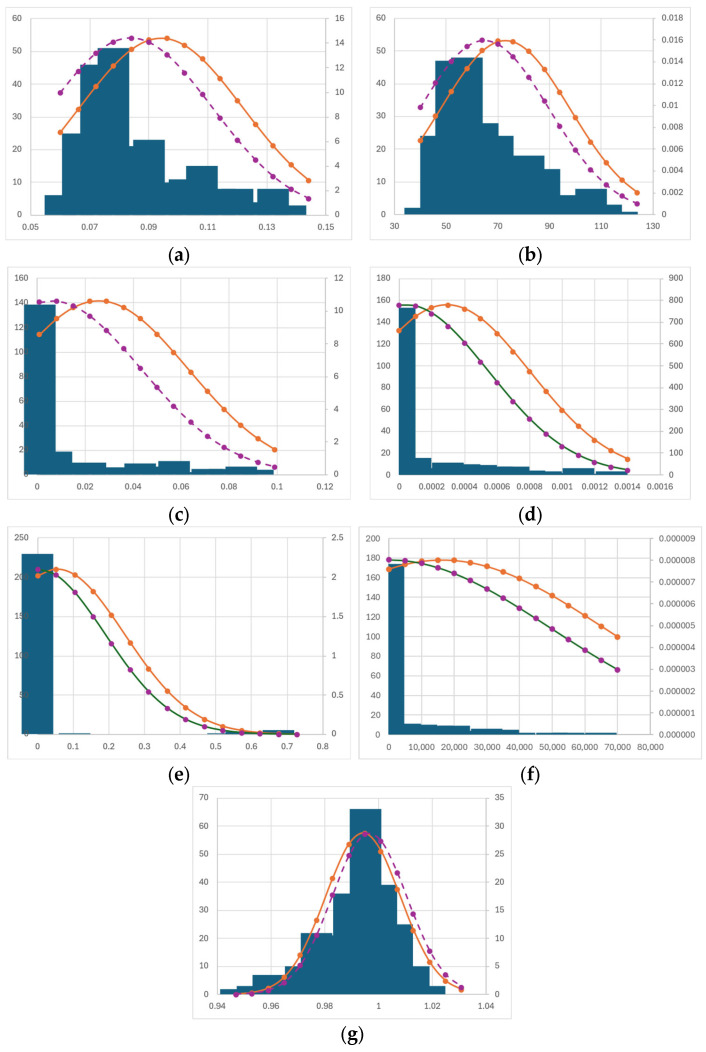
Histograms and the normal distribution for all the variables of Model D. The dashed lines correspond to the normal distribution when the median is taken as the mean value in the normal distribution. (**a**) *µ_max_* (**b**) *K_I_* (**c**) *Q_min_* (**d**) *Q_max_* (**e**) *K_S_* (**f**) *Q_I_* (**g**) *α*.

For each model tested, bootstrapping was applied to all fitting parameters to obtain information on the uncertainty involved in the different model parameters. Since the number of measurements was high, the confidence interval was taken as follows:

(19) Confidence Interval=±2·σ

for a confidence level of 95%, where *σ* is the standard deviation. Below are the averages and medians for each model variable, as well as the respective standard deviations, and the confidence interval for each parameter (Table 4, Table 5, Table 6 and Table 7). The corresponding histograms for each variable are also presented for each model. The histograms for Model H, the best performing one, are presented in the main text (Figure 4), and the ones for the other models are provided in Appendix D (Figure A4, Figure A5 and Figure A6).

#### Bootstrapping Discussion

The obtained distributions for the bootstrapped data for Models M, H, and E show that the obtained mean values for each variable are in good agreement with the values obtained with the neutral weighting of the errors for each data point (that is, all errors with a weight of 1). This is confirmed by the fact that the parameters calculated with neutral weighting usually fall well within the confidence intervals obtained via bootstrapping.

The frequency distribution is often not symmetrical, which is due to the non-linear nature of the models tested. For the same reason, several of the parameters from Model D (namely *Q_min_*, *Q_max_*, *K_S_*, and *Q_I_*) show a very skewed distribution with many large outliers. This is again due to the non-linear nature of the systems but also to the fact that this model does not seem to be able to provide an adequate description of the biological processes. In fact, we can see that, for this model, several parameters have very wide confidence intervals. For example, the parameter *K_S_* is estimated as 72.86 ± 50 MJ m^−2^ day^−1^, which is a very large value when compared to Ks estimated by Model M, which is 17.73 ± 8.8 MJ m^−2^ day^−1^. This reinforces the idea that the model incorporating the evolution of nutrients needs a much wider revision.

## 4. Conclusions

In this article, four different growth models were applied to a dataset of ten assays of *Nannochloropsis* sp. with the objective of obtaining a model that would allow the description of the full set of experiments with a single set of kinetic parameters. The development of these models was carried out using a set of training assays to fit the model and a set of validation assays to test the validity of the model.

The results obtained show that a single growth model can be applied to all the different configurations that were tested with adequate predicting capabilities, as shown by the results in the validation set. The fact that well-established models in the literature were applied successfully, and that the results are supported by the validation assays, supports the validity of applying these models to pilot-scale cultivations.

Although most models performed well, overall, it was concluded that the best model among the ones tested is Model H, which has the lowest AIC and overall lowest RMSE for both the training and the validation assays. This is also consistent with the narrower confidence intervals obtained by the bootstrapping method.

This result lends itself to the conclusion that this microalgae species has an ideal growth irradiation of around 41 MJ m^−2^ day^−1^, after the effects of light attenuation are taken in consideration, with the growth rate diminishing at higher irradiations. However, Models M and E also have very similar results, which suggests they could be adequately used to predict future cultivations.

Model D, which takes into account nutrient concentration, performed the worst of all the models. This implies that modeling these systems with the effect of nutrients not only is not needed to explain most of the growth, under the conditions that were used in these cultivations, but can even be a hindrance. Another possibility is that, since nutrients were given in excess, the influence of their effect on growth was minimal.

Future work should involve further tests of the models using new cultivations of the same species in similar and different conditions to ascertain the predictive range of this model.

## Figures and Tables

**Table 1:** Assay codes used and duration of each assay.

Assay Code	Duration (Days)	Number of Data Points	Assay Type
M1-1	41	15	Validation
M1-2	95	38	Training
M1-3	19	17	Validation
M1-4	117	38	Training
U1-1	113	44	Training
U1-2	33	16	Training
U1-3	82	25	Validation
U2-1	113	42	Validation
U2-2	20	14	Training
U2-3	33	17	Validation

**Table 2:** RMSEs resulting from the fitting of the five training assays, along with the global R^2^.

	RMSE			
Assay	Model M	Model H	Model E	Model D
M1-2	0.070	0.079	0.102	0.170
M1-4	0.107	0.091	0.082	0.217
U1-1	0.104	0.106	0.098	0.161
U1-2	0.121	0.080	0.097	0.120
U2-2	0.073	0.048	0.054	0.130
Global	0.098	0.090	0.093	0.173

**Table 3:** RMSEs resulting from the fitting of the five validation assays.

	RMSE			
Assay	Model M	Model H	Model E	Model D
M1-1	0.295	0.240	0.380	0.951
M1-3	0.360	0.407	0.273	0.236
U1-3	0.428	0.383	0.405	0.235
U2-1	0.206	0.220	0.211	0.292
U2-3	0.245	0.206	0.227	0.131
Global	0.309	0.296	0.302	0.413

**Table 4:** Mean, median, standard deviation, confidence interval, and the value obtained from the fitting with the full dataset for every parameter of Model M.

Parameter	Mean	Median	St. Dev.	Confidence Interval	Full Dataset Fitting
*µ_max_* (h^−1^)	0.045	0.044	0.005	±0.01	0.041
*K_S_* (MJ m^−2^ day^−1^)	17.13	16.95	4.404	±8.8	13.3
*α* (m^3^ kg^−1^)	1.555	1.528	0.133	±0.26	1.645

**Table 5:** Mean, median, standard deviation, confidence interval, and the value obtained from the fitting with the full dataset for every parameter of Model H.

Parameter	Mean	Median	St. Dev.	Confidence Interval	Full Dataset Fitting
*µ_max_* (h^−1^)	0.033	0.033	0.001	±0.001	0.032
*I_opt_* (MJ m^−2^ day^−1^)	41.74	40.84	5.549	±11.1	41.4
*γ*	1.106	1.063	0.214	±0.428	1.070
*α* (m^3^ kg^−1^)	1.399	1.385	0.093	±0.186	1.337

**Table 6:** Mean, median, standard deviation, confidence interval, and the value obtained from the fitting with the full dataset for every parameter of Model E.

Parameter	Mean	Median	St. Dev.	Confidence Interval	Full Dataset Fitting
*µ_max_* (h^−1^)	0.034	0.034	0.002	±0.004	0.033
*λ*_2_ (MJ m^−2^ day^−1^)	10.13	9.957	1.631	±3.26	9.933
*α* (m^3^ kg^−1^)	1.527	1.528	0.113	±0.22	1.515

**Table 7:** Mean, median, standard deviation, confidence interval, and the value obtained from the fitting with the full dataset for every parameter of Model D.

Parameter	Mean	Median	St. Dev.	Confidence Interval	Full Dataset Fitting
*μ_max_* (h^−1^)	0.094	0.084	0.028	±0.056	0.082
*K_S_* (MJ m^−2^ day^−1^)	72.86	64.74	25.02	±50	63.3
*Q_min_* (g_N_ g_C_^−1^)	0.026	0.006	0.037	±0.074	0.0025
*ρ_max_* (g_N_ g_C_^−1^ h^−1^)	3 × 10^−4^	4 × 10^−5^	5 × 10^−4^	±1 × 10^−3^	8 × 10^−6^
*K_sub_* (g_N_ g_C_^−1^)	0.055	0	0.189	±0.378	0
*Q_I_* (g_N_ g_C_^−1^)	16334	14.84	49772	±99544	3.98
*α* (m^3^ kg^−1^)	0.994	0.997	0.014	±0.28	1.003

**Figure A1:**
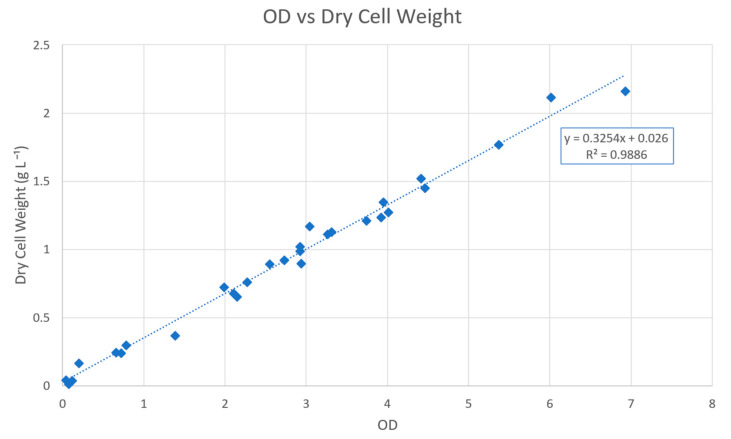
Correlation between measured OD and the dry cell weight for *Nannochloropsis* sp. The adjusted parameters were a slope of 0.3254 ± 0.0067 g L^−1^ and an intercept of 0.026 ± 0.022 g L^−1^.

**Figure A2:**
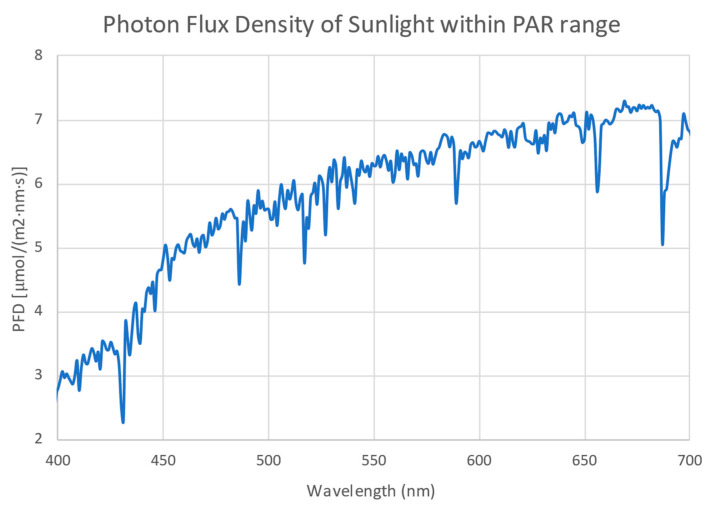
Solar spectrum within the PAR, in units of photon flux density. Adapted from [35].

**Figure A3:**
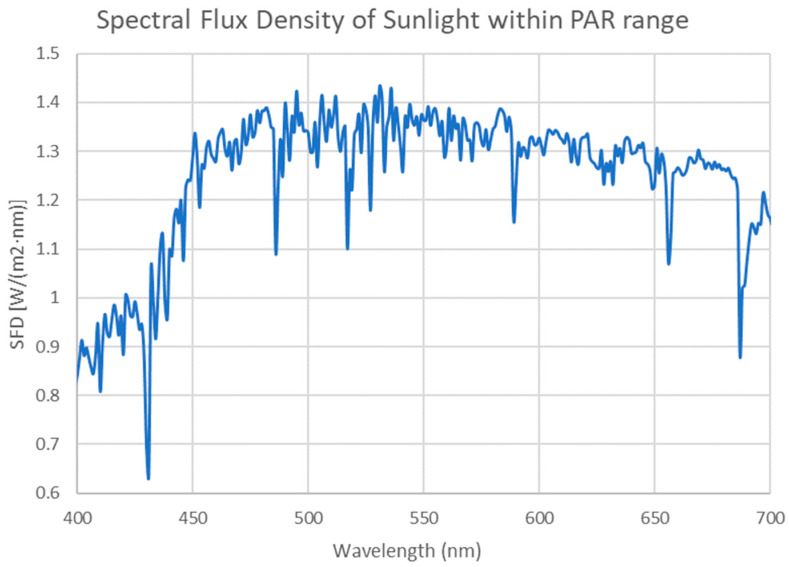
Spectral flux density of sunlight within the PAR. Adapted from [35].

## Data Availability

The datasets presented in this article are not readily available because they are under a non-disclosure agreement with A4F. Requests to access the datasets should be directed to the corresponding author.

## Appendix A

The dry cell weight (DCW) of the assays was measured indirectly using a correlation with the optical density (OD) of the culture. This correlation for *Nannochloropsis* sp. measured at 750 nm is found in Figure A1.

The DCW measured to construct this correlation was done using the following protocol. First, three microfiber filters (with a 0.7 μm pore size) are dried and weighed using a moisture balance, with their combined weight being registered (*m_i_*). A certain volume of culture is filtered through each microfiber filter using a vacuum filter, with the filtered volume *V* being registered. The three filters are then dried and weighed together in the moisture balance (*m_f_*). The dry cell weight (*DCW)* is given by Equation (A1):

(A1) $$DCW=\frac{m_{f}-m_{i}}{V}$$

## Appendix B

Starting from Eilers and Peeters’ formula,

(A2) $$\mu =\mu_{max}\left(1+\beta \right)\frac{2x_{I}}{x_{I}^{2}+2\;\beta x_{I}+1}$$

Replacing 1 + *β* with a new variable *γ*, and the variable *x_i_* with *I*/*I_opt_*,

(A3) $$\mu =\mu_{max}\gamma \frac{2\frac{I}{I_{opt}}}{{\left(\frac{I}{I_{opt}}\right)}^{2}+2\;\left(\gamma -1\right)\frac{I}{I_{opt}}+1}↔\frac{2\mu_{max}\gamma I}{{\frac{I}{I_{opt}}}^{2}+2\;I\left(\gamma -1\right)+I_{opt}}↔\;↔\frac{2\mu_{max}\gamma I\cdot I_{opt}}{I^{2}+2\;\gamma I\cdot I_{opt}-2\;I\cdot I_{opt}+{I_{opt}}^{2}}↔\frac{2\mu_{max}\gamma I\cdot I_{opt}}{\;2\;\gamma I\cdot I_{opt}+{\left(I-I_{opt}\right)}^{2}}↔\;↔\frac{\mu_{max}\cdot k}{\;k+{\left(I-I_{opt}\right)}^{2}},\;k=2\;\gamma I\cdot I_{opt}$$

## Appendix C

In all modeling equations involving microalgae, the light energy emitted by the light source (be it the sun or artificial light) is usually denoted as *I*_0_. However, there are two different but complementary ways to describe *I*_0_—as the total number of photons passing through a certain area (photon flux density, or *PFD*, in units of mol_photons_ m^−2^ s^−1^), or as the total amount of energy carried by those photons (radiance, W m^−2^). A closely related quantity to radiance is spectral flux density (*SFD*), a measure of the energy transferred by electromagnetic radiation of a certain wavelength through a certain area; the unit is called the Janksy, or W m^−2^ nm^−1^. *SFD* is defined per wavelength, while *PFD* is usually defined within a certain range, like PAR (Photosynthetic Active Range).

*I*_0_ can thus be defined as the photon flux density of the PAR, and can be calculated with Equation (A4):

(A4) $$I_{0}={PFD}_{PAR}=\int_{400}^{700}{PFD}_{\lambda }d\lambda$$

where *PFD_PAR_* is the photon flux density in the PAR region of the spectrum and *PFD_λ_* is the spectral flux density for each wavelength.

The photon flux density of sunlight within the PAR is illustrated in Figure A2.

On the other hand, *I*_0_ can also be the radiance of the PAR, that is, the total amount of energy transmitted. The *SFD_λ_*, or the amount of energy transmitted by a single wavelength, can be calculated from the *PFD_λ_* using the Planck relation [36]:

(A5) $${SFD}_{\lambda }={PFD}_{\lambda }\times h\cdot v{\cdot N}_{A}={PFD}_{\lambda }\times \frac{h\cdot c{\cdot N}_{A}}{\lambda }$$

with *h* being the Plank constant (6.62607015 × 10^−34^ J⋅s), *SFD_λ_* the spectral flux density of a given source of light, *N_A_* the Avogadro’s constant (6.02214076 × 10^23^), *ν* the photon frequency, *c* the speed of light, and *λ* the wavelength.

The radiance is thus the integral of all *SFD_λ_* over the band of interest (in this case, PAR):

(A6) $$I_{0}={SFD}_{PAR}=\int_{400}^{700}{SFD}_{\lambda }d\lambda$$

$$I_{0}=\int_{400}^{700}\frac{h\cdot c{\cdot N}_{A}}{\lambda }\times {PFD}_{\lambda }d\lambda =h\cdot c{\cdot N}_{A}\int_{400}^{700}\frac{{PFD}_{\lambda }}{\lambda }d\lambda$$

The spectral flux density of sunlight within the PAR is illustrated in Figure A3.

The difference between these two quantities is essentially the difference in energies transmitted by each wavelength of light: longer wavelengths transmit less energy, as per the Planck relation. Which of these quantities should be used in microalgae modeling depends on whether distinguishing different wavelengths is important or not. *SFD* is potentially more useful when the important factor for the modeling is the total energy absorbed by the culture. On the other hand, *PFD* is more useful when the effects of each individual wavelength are considered since it counts how many photons of a certain wavelength hit the reactor and not their energy.

The conversion between one quantity and the other depends on the source of light used. If one is comparing different light sources, it is advisable to convert to the unit whose light source’s wavelength distribution is not known.

Converting between *PFD_PAR_* and *SFD_PAR_* for sunlight is equivalent to calculating the average energy of one mole of photons of sunlight, or *E_γPAR_*. That value can be calculated by dividing *SFD_PAR_* by *PFD_PAR_*.

(A7) $$E_{\gamma PAR}=\frac{{SFD}_{PAR}}{{PFD}_{PAR}}$$

Since [35] gives the values in *SFD_Φ_*, to calculate *SFD_PAR_* it is only necessary to integrate the values in the PAR. To calculate *PFD_PAR_* it is necessary to solve Eq.C.3 for each wavelength and then integrate in the PAR. Using this method, *SFD_PAR_* is equal to 375.8 W m^−2^ and *PFD_PAR_* is equal to 1740 μmol m^−2^ s^−1^. Therefore, by solving Equation (A7), *E_γPAR_* is equal to 0.2160 J μmol^−1^.
